# Single-layer low-profile high-gain wideband circularly polarized MIMO antenna for UAV applications

**DOI:** 10.1371/journal.pone.0362484

**Published:** 2026-11-13

**Authors:** Hung Nguyen-Manh, Giang Nguyen-Hoai, Thai Nguyen-Dinh, Duy-Phong Pham, Nguyen Quoc Dinh

**Affiliations:** 1 Faculty of Electrical and Electronics Engineering, Hanoi Open University, Hanoi, Vietnam; 2 Faculty of Electronics and Telecommunications, Electric Power University, Hanoi, Vietnam; 3 Faculty of Electrical and Electronic Engineering, PHENIKAA School of Engineering, PHENIKAA University, Hanoi, Vietnam; 4 Advanced Wireless Communications Group, Le Quy Don Technical University, Hanoi, Vietnam; Galgotias College of Engineering and Technology, Greater Noida, INDIA

## Abstract

This paper presents a single-layer circularly polarized (CP) multiple-input multiple-output (MIMO) antenna with wideband operation and high-gain radiation characteristics. The antenna comprises 16 truncated-corner patches arranged in a 4×4 configuration and capacitively excited by a probe-fed rectangular patch, enabling a low-profile single-layer structure. The final two-port MIMO antenna has a low profile of approximately 0.02λ at the lowest operating frequency. The measured results demonstrate an effective operating bandwidth of 5.16–5.68 GHz, corresponding to 9.6%, with isolation better than 19 dB and a peak gain of 9.4 dBi. In addition, the antenna exhibits good diversity performance in terms of envelope correlation coefficient (ECC), channel capacity loss (CCL), and mean effective gain (MEG). These results confirm that the proposed antenna is a promising candidate for unmanned aerial vehicle (UAV) communication systems.

## Introduction

The extensive use of unmanned aerial vehicles (UAVs) has strongly influenced modern wireless communication systems across diverse civilian and commercial applications [1]. Reliable, high-data-rate, and long-range links are essential for these systems. Accordingly, multiple-input multiple-output (MIMO) antennas are widely used to enhance transmission capacity and spectral efficiency without requiring additional bandwidth [2–4]. Moreover, high-gain antennas are desirable for long-range UAV communications because they improve the link budget, compensate for free-space path loss, and extend coverage. However, UAV flight motions, such as pitching and rolling, can cause polarization mismatch when linearly polarized antennas are used. Circularly polarized (CP) radiation is therefore preferred to reduce polarization loss and suppress multipath fading [5]. Hence, this paper focuses on a high-gain, wideband CP MIMO antenna for reliable long-range UAV communication.

Monopole and slot antennas [6–10] are promising candidates for low-profile wideband CP MIMO antennas. However, owing to their inherent bidirectional radiation characteristics, they usually exhibit relatively low gain and are less attractive for applications requiring high-gain unidirectional radiation. In particular, UAV-to-ground-station communication generally benefits from a unidirectional beam to concentrate radiated energy toward the ground station, rather than a bidirectional radiation pattern.

Microstrip patch antennas are promising candidates for generating directional CP beams while maintaining a low-profile and lightweight structure [11–15]. These designs are typically based on single-layer configurations, but they generally provide narrow CP operating bandwidths, usually less than 3%, and moderate gain, typically around 7 dBi. High isolation is commonly achieved by employing defected ground structures (DGSs) or grounded stubs. Wider bandwidth can be obtained by using additional radiating elements [16,17], power dividers [18,19], or frequency-selective surfaces (FSSs) [20,21]. However, these approaches often require multilayer configurations, which increase the structural complexity and profile. Moreover, some designs still exhibit limited gain, usually below 9 dBi, and/or high back radiation due to the use of DGS-based decoupling networks.

Wideband CP operation with a single-layer configuration has been reported in [22–25], where CP bandwidths greater than 12% can be achieved. In these designs, isolation is mainly improved through electromagnetic-field redistribution using parasitic elements or metasurface layers. Nevertheless, their gain is typically lower than 9 dBi, which may be insufficient for long-range UAV communication systems.

To conclude, for long-range UAV communication, antennas should provide circular polarization, high directional gain, wide bandwidth, and a low-profile structure. However, existing CP MIMO antennas often suffer from low gain, narrow bandwidth, or increased complexity due to multilayer structures and additional decoupling or feeding networks. Therefore, the motivation of this work is to simultaneously achieve wide CP bandwidth, high gain, low profile, and adequate MIMO isolation. The main radiating element consists of sixteen truncated-corner microstrip patches arranged in a 4×4 configuration, which are capacitively excited by a centrally located rectangular patch. This configuration enables both high-gain radiation and wideband operation while maintaining a single-layer structure. By placing two radiating elements in close proximity, a two-port MIMO antenna is realized. The measured results show that the proposed antenna achieves a wide CP operating bandwidth of 9.6%, with isolation better than 19 dB and a peak gain of 9.4 dBi. In addition, the antenna exhibits good diversity performance, confirming its suitability for long-range UAV communication systems.

The contribution of the work lies in the combination of the following features within one planar layer: (i) a centrally excited 4 × 4 truncated-corner patch aperture for high gain; (ii) multiple slightly different CP resonators that create adjacent resonances and broaden the CP operating band; (iii) capacitive excitation that avoids a multi-layer corporate feed; and (iv) a two-port MIMO configuration achieving comparable measured performance. The contribution is therefore the simultaneous realization of wideband CP, high gain, low profile, and simple MIMO integration rather than any single metric alone.

## Wideband high-gain CP antenna

Existing monopole/slot CP MIMO antennas are generally low gain and bidirectional, whereas conventional patch-based CP MIMO antennas usually provide narrow CP bandwidth. Wider-band and higher-gain solutions often rely on stacked layers, superstrates, power dividers, or DGSs, which increase the profile, structural complexity, or back radiation. Thus, the motivation of this work is to simultaneously satisfy four requirements that are particularly relevant to long-range UAV links: wide CP bandwidth, high directional gain, low-profile single-layer implementation, and MIMO operation with adequate isolation.

The selected resonator arrangement is motivated by the need to enhance both CP bandwidth and gain without introducing additional layers or a corporate feeding network. A truncated-corner patch supports the two near-degenerate orthogonal modes required for CP radiation. By incorporating additional truncated-corner patches with slightly different resonant dimensions, a second CP resonance is introduced at a nearby higher frequency, thereby broadening the 3-dB axial-ratio bandwidth. Extending this concept to a 4 × 4 capacitively coupled aperture enlarges the effective radiating aperture and improves the broadside gain, while the central rectangular feed maintains a single-layer configuration. The design evolution of the proposed wideband high-gain CP antenna is discussed in the following sub-sections.

### Design-1

Fig 1 demonstrates a CP antenna, denoted as Design-1. The antenna is designed on a Taconic RF-35 dielectric substrate with a relative permittivity of 3.5, a loss tangent of 0.0018, and a thickness of 1.52 mm. The main radiator is a simple square patch with dimensions of 13.2 mm × 13.2 mm. To create orthogonal modes for CP radiation, geometrical deformations are introduced through truncation of the corners by 3.3 mm. The CP antenna is excited with the help of a 50-Ω SMA feedline. Further demonstration about the operating principle of this antenna can be found in [26]. Electromagnetic characteristics were investigated with the use of full-wave electromagnetic modeling by HFSS. The optimized dimensions of the antenna are represented in millimeters (unit: mm) as follows: $W_{s}=55$, *w* = 12.5, $l_{c}=3.0$, *s* = 0.3, *g* = 0.12, $l_{o}=14$, $w_{o}=2.2$, $l_{f}=6$.

**Fig 1:**
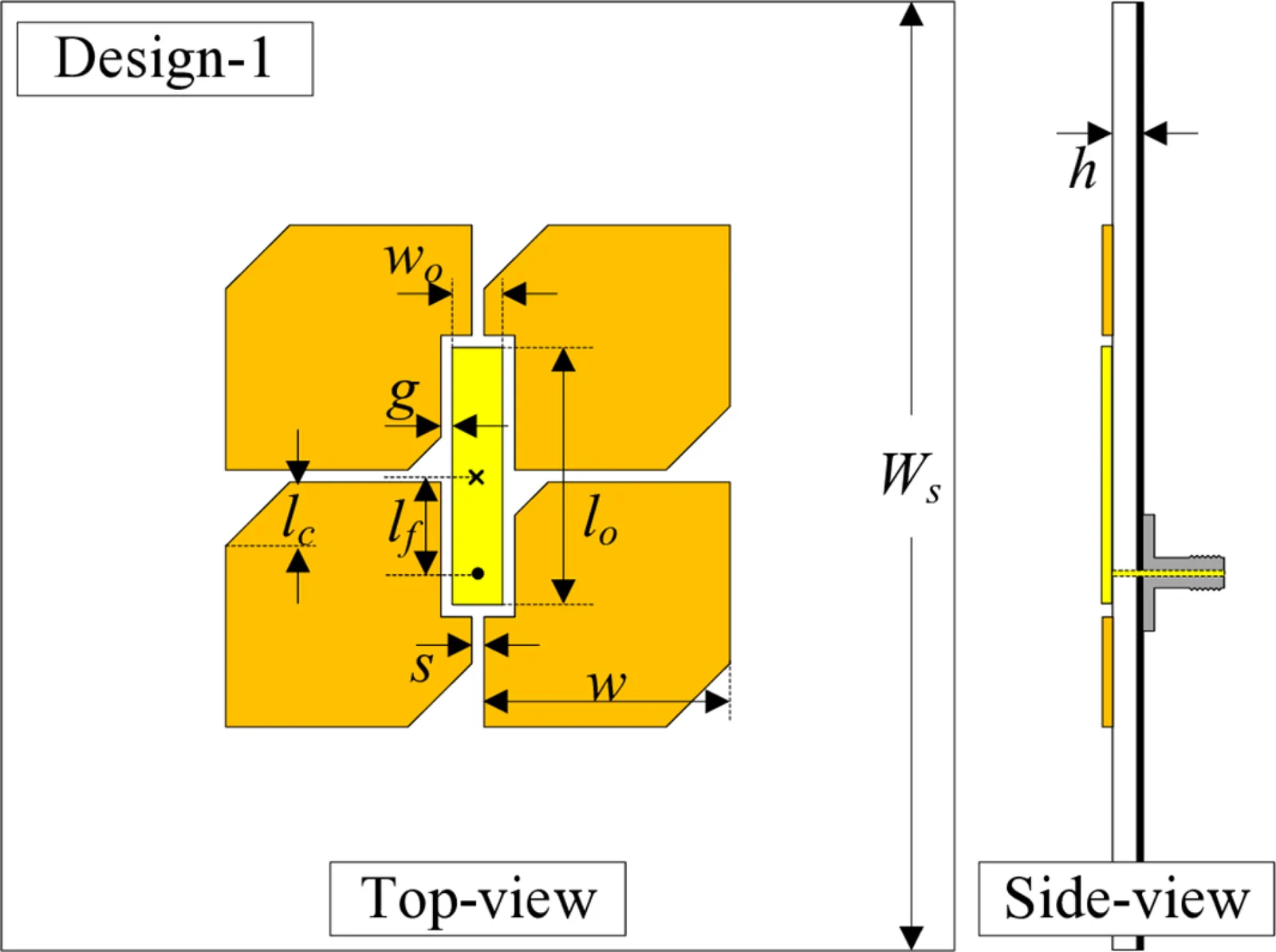
Geometry of Design-1.

As demonstrated in Fig 2, the simulated reflection coefficient shows a great impedance matching ability as the |*S*_11_| parameter stays below the threshold of −10 dB in the frequency band from 4.88 to 5.66 GHz. Simultaneously, 3-dB AR BW is achieved within the range from 5.17 to 5.31 GHz. Intersection of these two ranges defines the working BW as 2.7% around the center frequency of 5.24 GHz. The realized gain within this working frequency range is stable with the maximum value of about 8.0 dBi observed at the center frequency.

**Fig 2:**
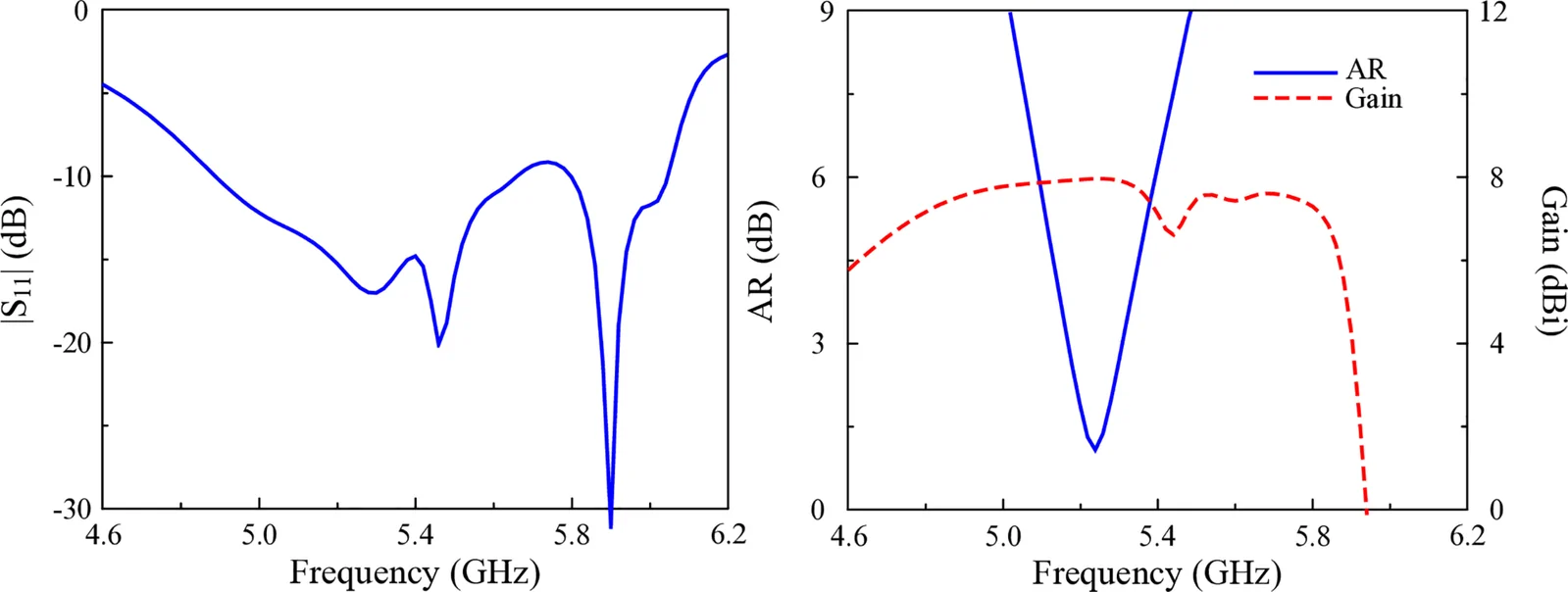
Simulated |*S*_11_|, AR, and broadside gain of Design-1.

### Design-2 with enhanced AR BW

Fig 3 illustrates the evolution from Design-1 to Design-2. For AR BW enhancement, additional truncated corner patches with different sizes are utilized. These additional patches are used to produce additional AR resonance in high frequency region. The optimized values for Design-2 are: $W_{s}=55$, *w* = 12.2, $l_{c}=3.2$, *s* = 0.3, *g* = 0.12, $l_{o}=12.5$, $w_{o}=2.6$, $l_{f}=3.8$, *w*_1_ = 12.0, $l_{c1}=3.2$, *s*_1_ = 0.3 (unit: mm).

**Fig 3:**
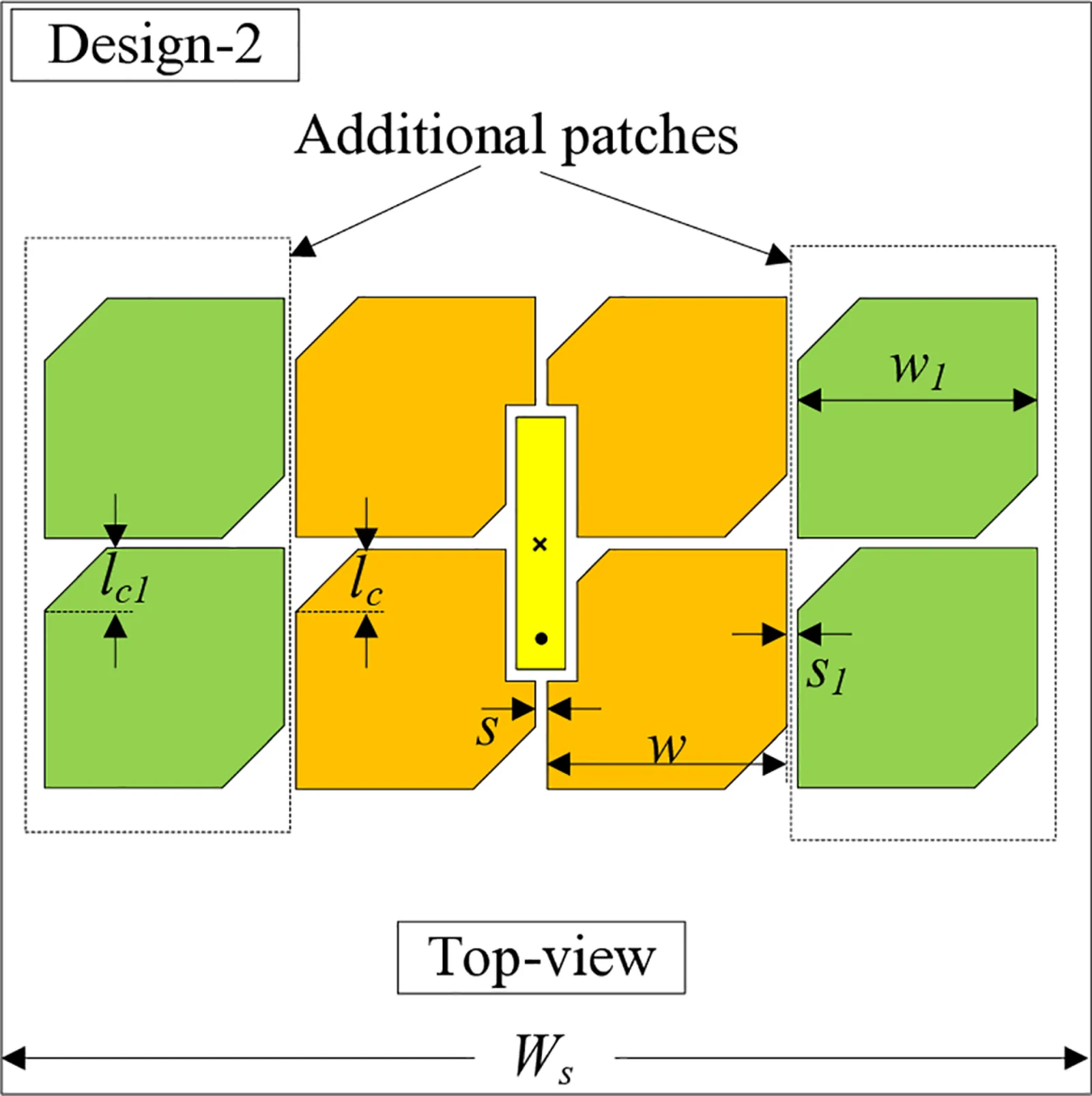
Geometry of Design-2 with enhanced AR BW.

Fig 4 shows the simulated AR performance of Design-2 with different sizes of the additional patches, *w*_1_. It can be seen obviously that changing *w*_1_ only has a strong effect on the AR resonance in the high frequency range, which shifts to a lower band when increasing *w*_1_. Meanwhile, the lower band remains unchanged. The reason behind this is that this resonance is strongly produced by the four center patches.

**Fig 4:**
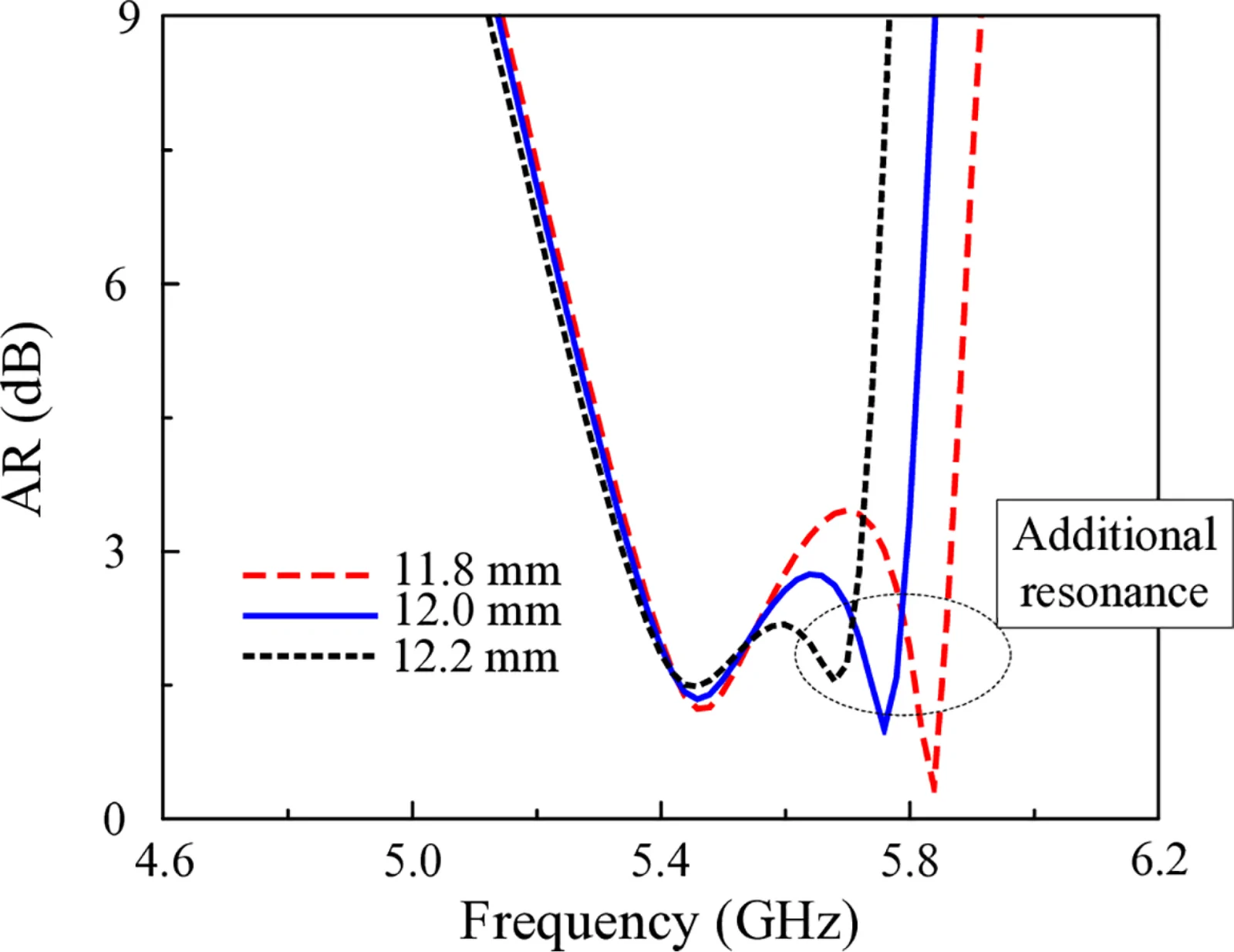
Simulated AR of Design-2 with different values of *w*_1_.

### Design-3 with enhanced AR BW and gain radiation

Fig 5 illustrates the evolution from Design-2 to Design-3. For gain improvement, the radiating aperture is increased by incorporating multiple truncated corner patches around the four center patches. The optimized values for Design-3 are: $W_{s}=55$, *w* = 12.2, $l_{c}=3.2$, *s* = 0.3, *g* = 0.12, $l_{o}=12.5$, $w_{o}=2.6$, $l_{f}=3.8$, *w*_1_ = 12.0, $l_{c1}=3.2$, *s*_1_ = 0.3 (unit: mm).

**Fig 5:**
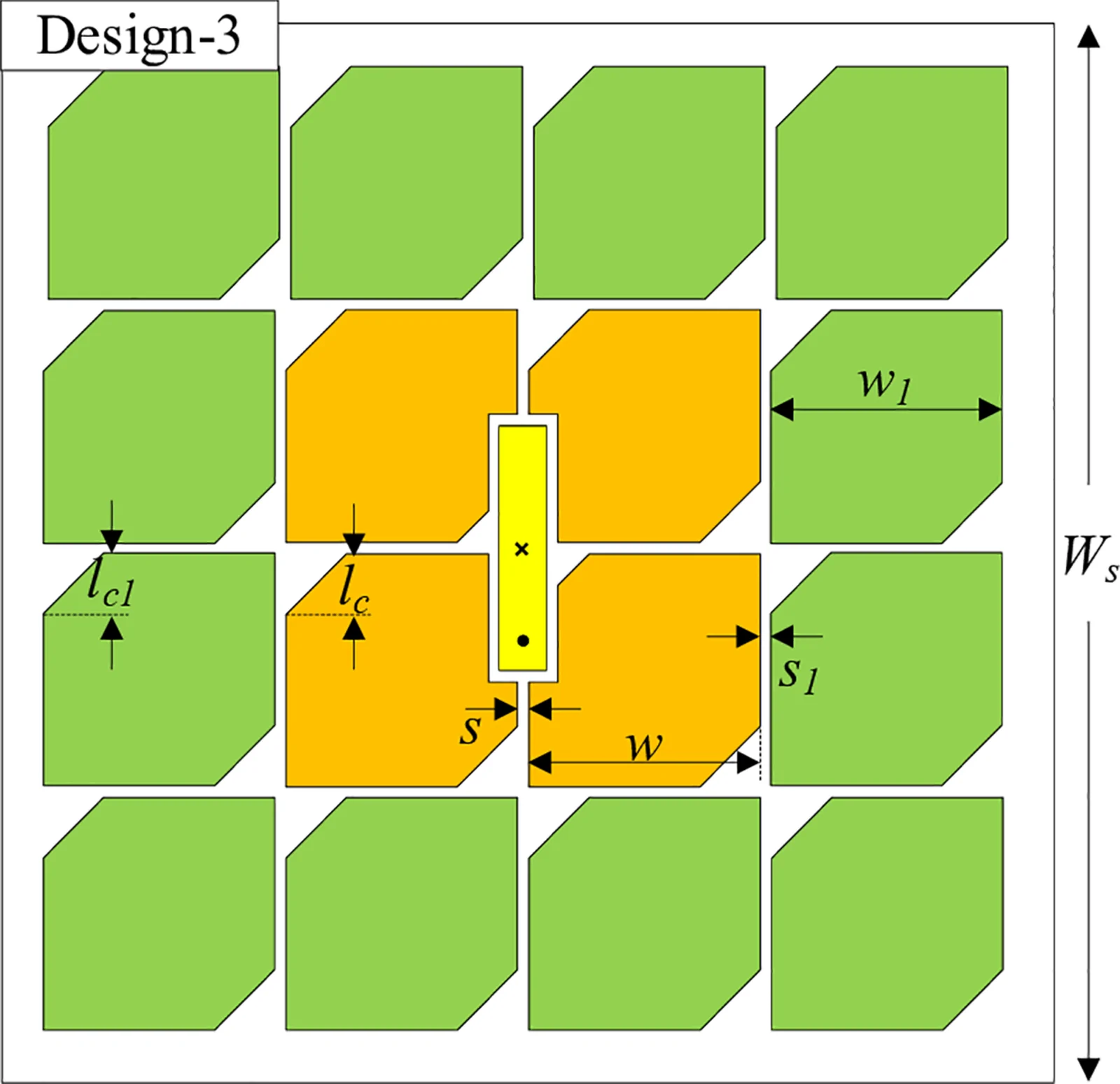
Geometry of Design-3 with enhanced AR BW and gain radiation.

Fig 6 shows the simulated AR and gain performance of different antennas. Obviously, Design-1 shows the smallest AR BW with single AR resonance. Meanwhile, Design-2 and Design-3 exhibit larger BW with the presence of an additional AR resonance in the high frequency range. In terms of gain, the increase in radiating aperture allows Design-3 to achieve the best gain performance. Compared to Design-1, the gain increases from about 8.0 to 9.8 dBi, the enhancement is about 1.8 dBi. It is worth noting that the overall dimensions of all designs are kept similar to 55 mm × 55 mm. Overall, the performance enhancement from Design-1 to Design-3 originates mainly from the capacitively coupled parasitic patch aperture, rather than from slots or a modified ground plane.

**Fig 6:**
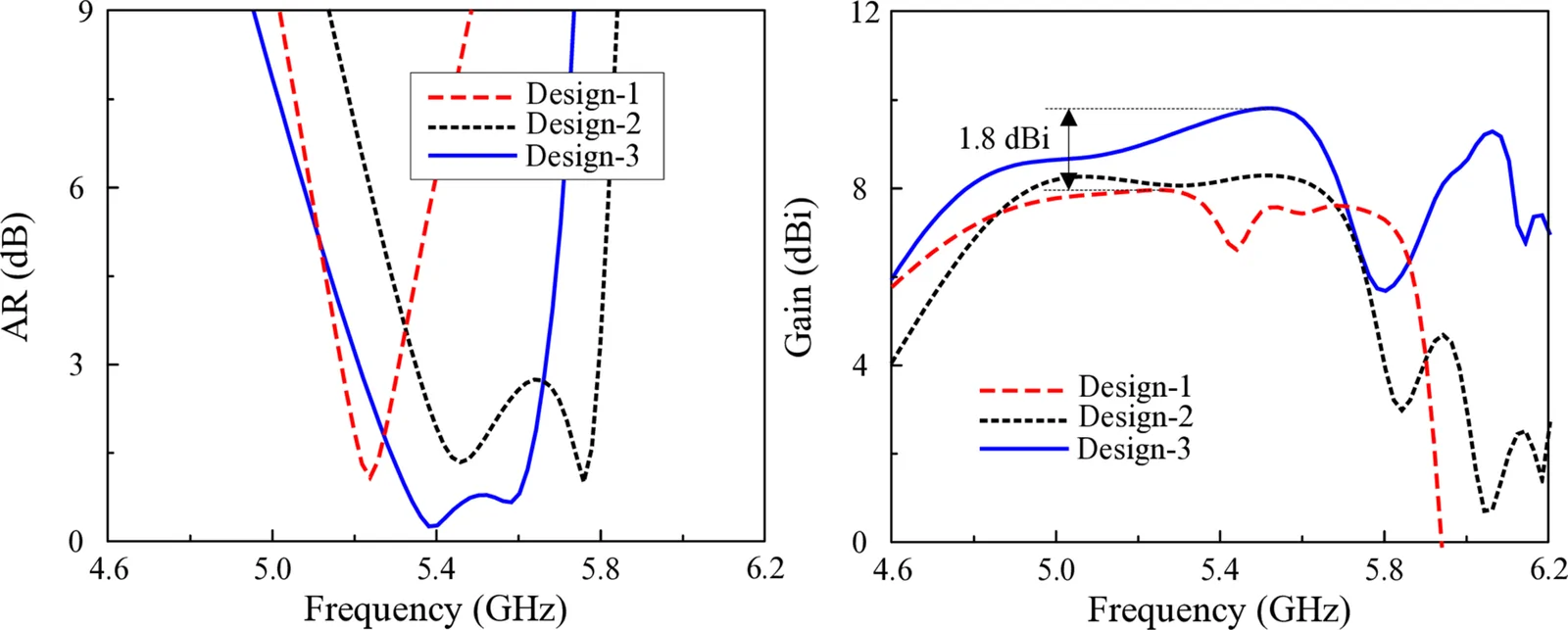
Simulated AR and broadside gain of different antennas.

The BW enhancement of the proposed Design-3 is first considered. Here, the wide CP bandwidth is realized by combining two nearby CP resonances. The four central truncated-corner patches mainly generate the lower CP resonance, whereas the surrounding patches, with slightly different dimensions, introduce an additional CP resonance at a higher frequency. The overlap of these resonances broadens the 3-dB AR bandwidth. Further demonstration can be obtained in Fig 7, which presents the current distributions of Design-3 at the two CP resonances, 5.3 and 5.5 GHz. At both frequencies, two orthogonal field components are observed, and the current rotates clockwise as the phase changes from $0^{\circ }$ to $90^{\circ }$, confirming LHCP radiation. In addition, the 5.3-GHz resonance is mainly associated with strong currents on the central patches, whereas the 5.5-GHz resonance shows stronger current distribution on the outer patches. This clearly demonstrates the role of the outer patches in generating the additional AR resonance.

**Fig 7:**
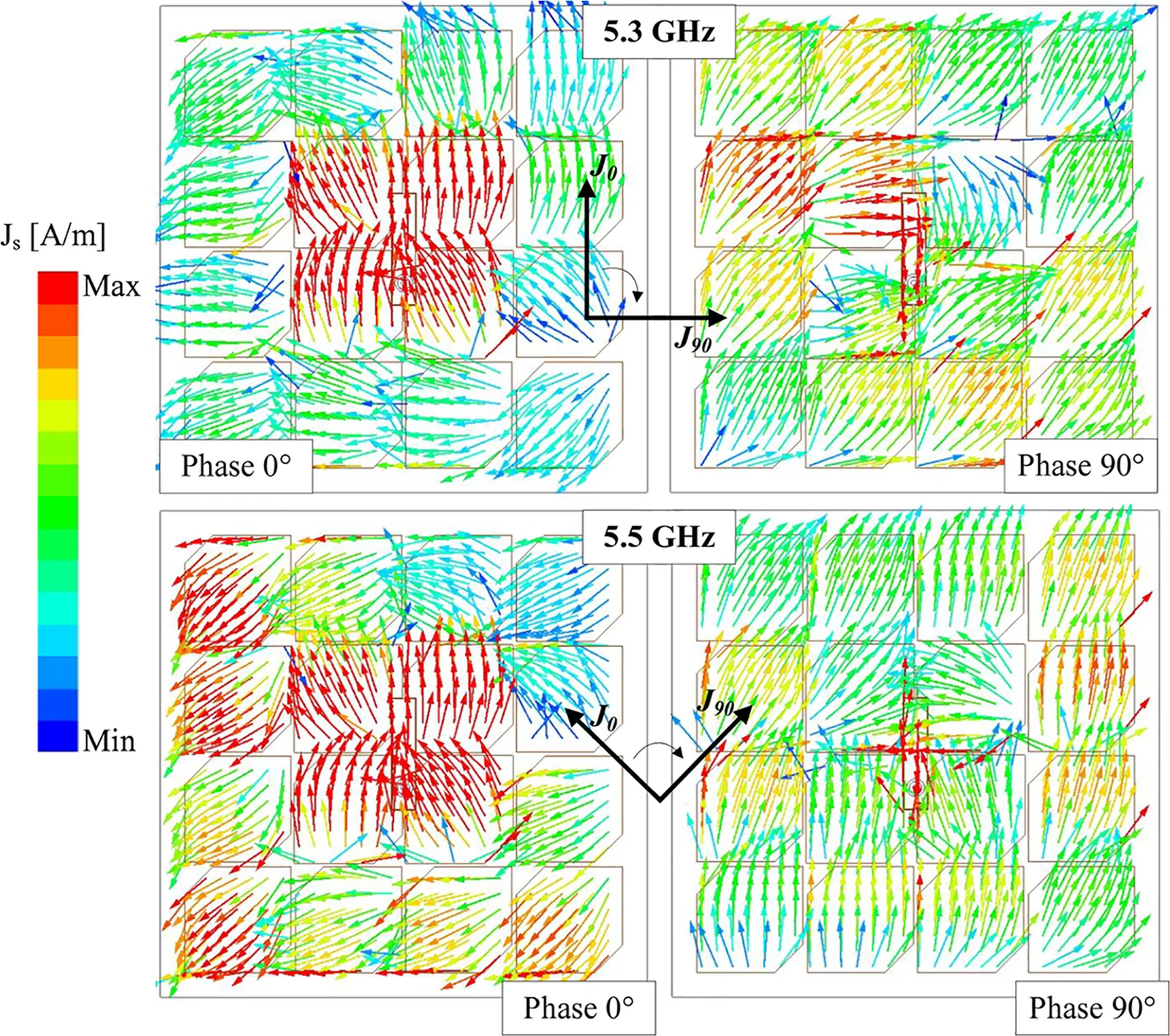
Simulated current distributions of Design-3 at different frequencies.

Regarding the gain performance, the gain enhancement is achieved by enlarging the actively radiating aperture. The antenna gain is related to the effective radiating aperture according to $G=4\pi A_{e}/\lambda^{2}$. Although Designs 1–3 have similar overall dimensions, Design-1 contains only the four central radiating patches, whereas Design-3 employs additional surrounding patches. The current distributions in Fig. 7 show that, at both CP resonances, significant currents are distributed over a larger portion of the aperture. Consequently, Design-3 provides a larger effective radiating region and achieves higher gain. This is also confirmed by the simulated results, where the gain increases from approximately 8.0 dBi for Design-1 to 9.8 dBi for Design-3. Further extension to a 5×5 arrangement provides only limited gain improvement because the outermost patches are weakly excited.

## 2-port wideband high-gain CP antenna

### Antenna geometry

The proposed final MIMO antenna design using the two-port Design-3 arrays is illustrated in Fig 8 where two such arrays have been placed together on one Taconic RF-35 dielectric substrate ($\varepsilon_{r}=3.5$, tanδ=0.0018, *h* = 1.52 mm). The antenna is excited by two 50-Ω SMA connectors. The overall dimensions of the antenna are 110 mm × 55 mm. The radiating arrays have been placed beside each other maintaining a center-to-center distance of $d_{e}=50$ mm. The optimized values for Design-3 are: $L_{s}=105$, $W_{s}=55$, *w* = 12.2, $l_{c}=3.2$, *s* = 0.3, *g* = 0.12, $l_{o}=12.5$, $w_{o}=2.6$, $l_{f}=3.8$, *w*_1_ = 12.0, $l_{c1}=2.8$, *s*_1_ = 0.3 (unit: mm).

**Fig 8:**
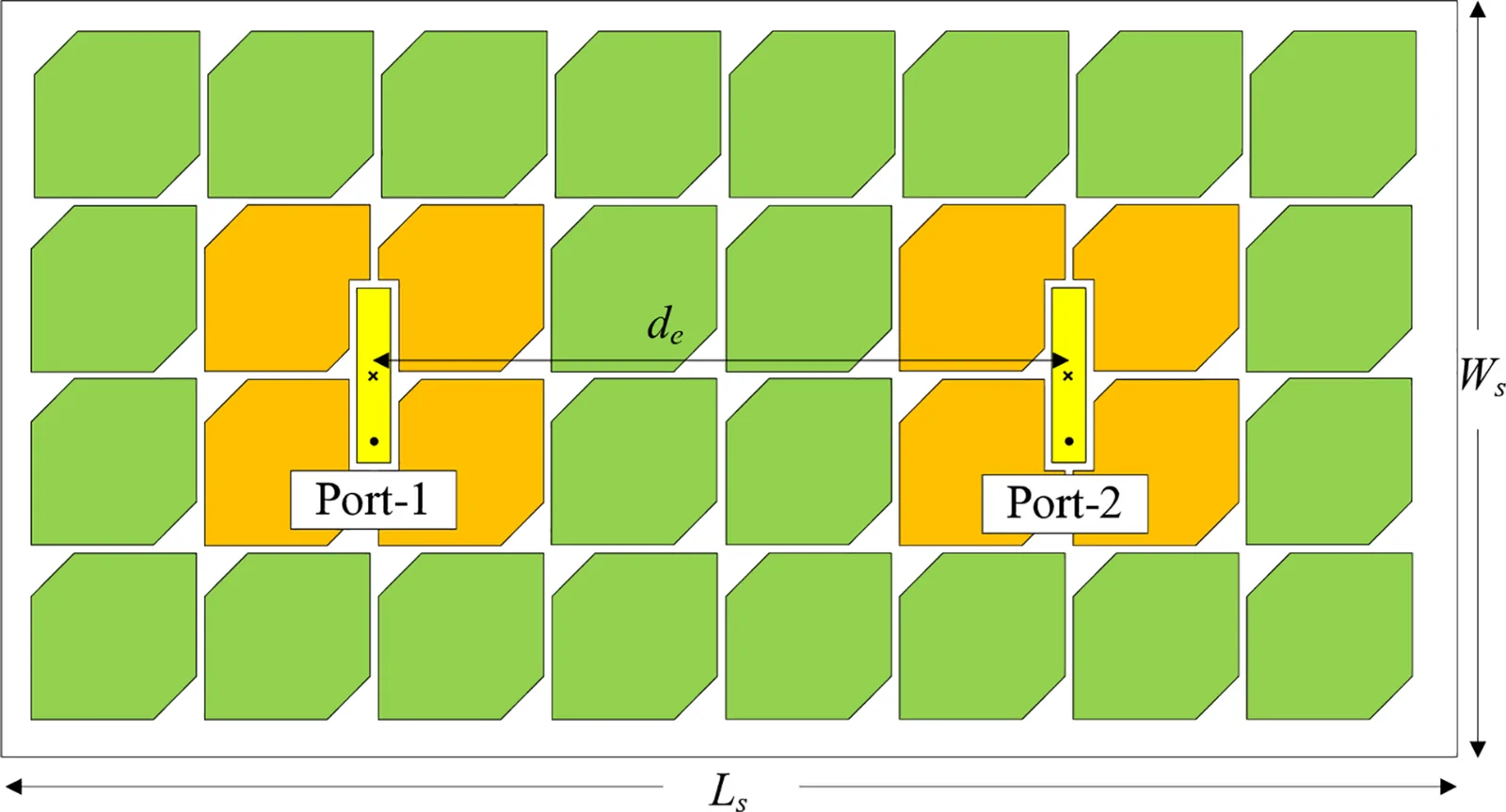
Geometry of the proposed MIMO antenna.

### Antenna operation characteristic

The simulated S-parameter in terms of reflection and transmission coefficients is shown in Fig 9. As seen, the antenna exhibits excellent impedance matching, remaining under −10 dB all the way from the start frequency 4.8 GHz to the end frequency 5.7 GHz for each antenna port. The 3-dB AR BW coincides with the impedance BW, resulting in a broad CP operating range of 9.3% (5.15–5.65 GHz). The MIMO configuration maintains an excellent mutual coupling of lower than 18 dB across the CP range. Besides, the gain varies from 6.0 to 9.6 dBi across the CP operating BW.

**Fig 9:**
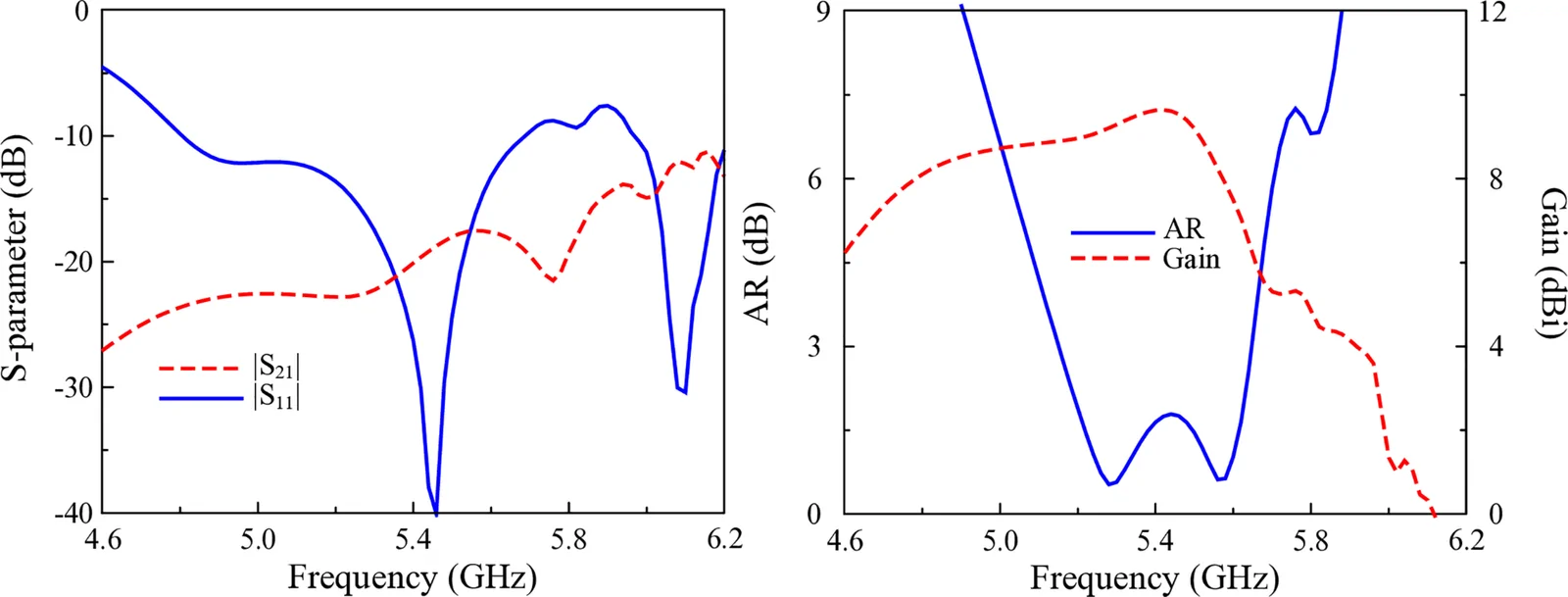
Simulated S-parameter, AR, and broadside gain of the proposed MIMO antenna.

Fig 10 shows the simulated transmission coefficient and gain of the proposed MIMO antenna for different element spacings, $d_{e}$. Increasing the element spacing leads to the reduction of mutual coupling between Port-1 and Port-2, resulting in better isolation. However, there is a trade-off with antenna size. In terms of gain, increasing the element distance slightly increases the antenna gain. The optimal value of $d_{e}$ is chosen as 50 mm to balance antenna performance and size.

**Fig 10:**
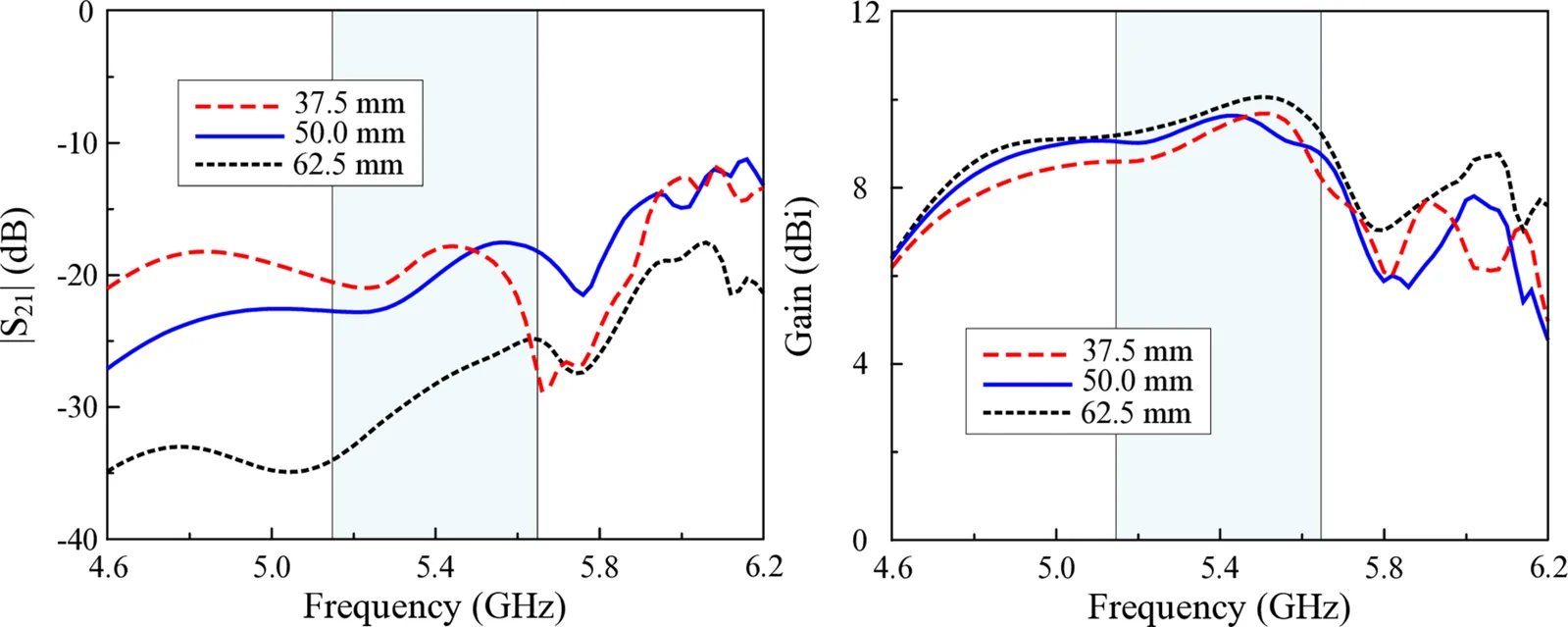
Simulated transmission coefficient and broadside gain of the proposed MIMO antenna for different element spacings ($d_{e}$).

### Measurement results

In order to verify the performance and characteristics of the proposed MIMO antenna by simulation results, a prototype was developed using conventional PCB fabrication methods. An illustration of the fabricated antenna is presented in Fig 11. The scattering parameters were measured through a Rohde & Schwarz ZNB20 vector network analyzer (VNA) with proper calibration.

**Fig 11:**
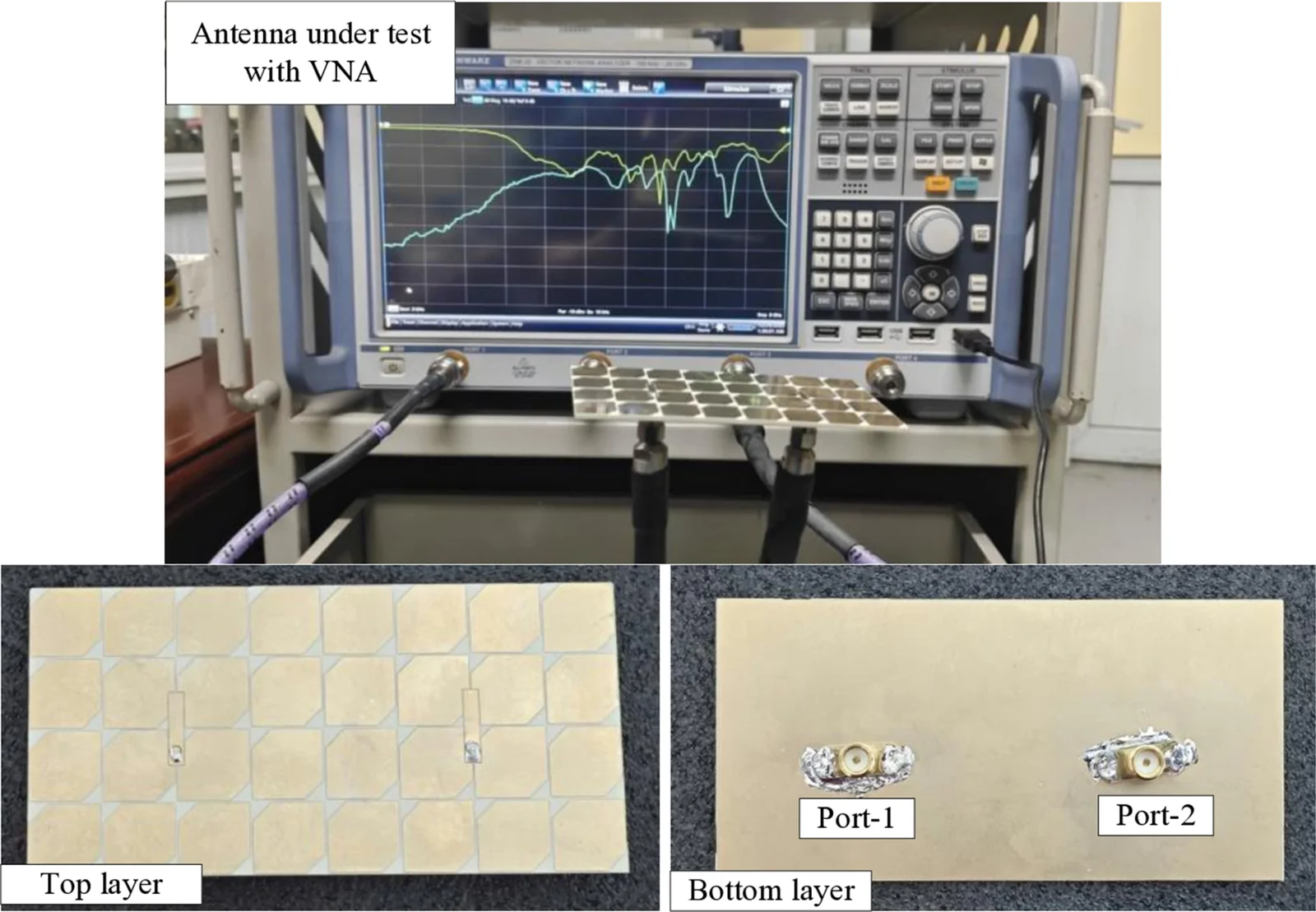
Photos of fabricated antenna and antenna under test with VNA.

### Antenna performance

Figs 12 and 13 show the simulated S-parameter and far-field radiation characteristics involving AR and broadside gain of the proposed antenna. It is observed that the fabricated prototype possessed a wide impedance matching BW, which fully covered the AR operating band from 5.16 to 5.68 GHz (approximately 9.6%). Across this band, the isolation is always better than 19 dB. The best isolation of 24 dB can be obtained around 5.3 GHz. Additionally, the measured peak realized gain also indicates that the antenna can achieve high gain from 7.6 to 9.4 dBi within the operating BW.

**Fig 12:**
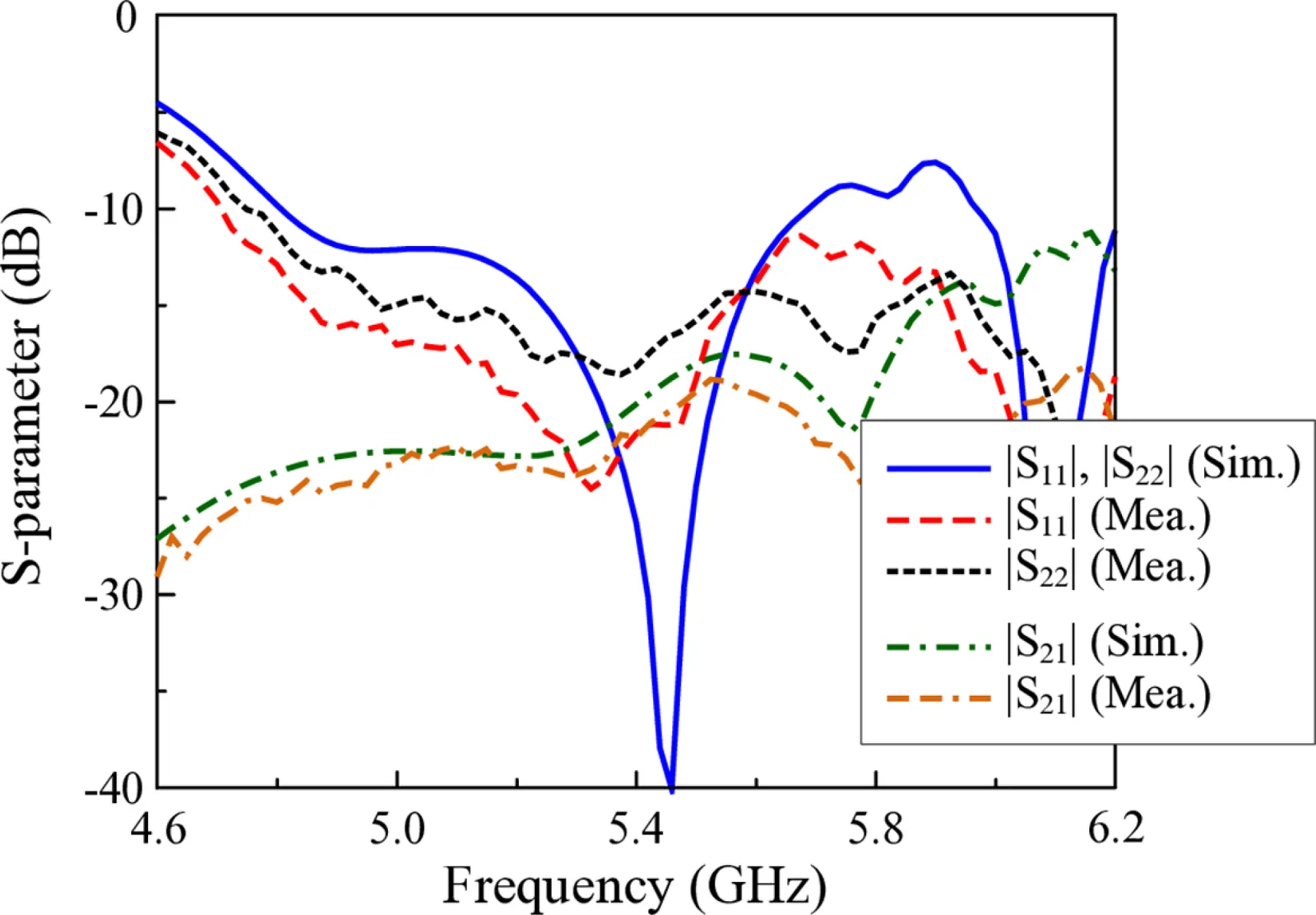
Simulated and measured S-parameter of the proposed antenna.

**Fig 13:**
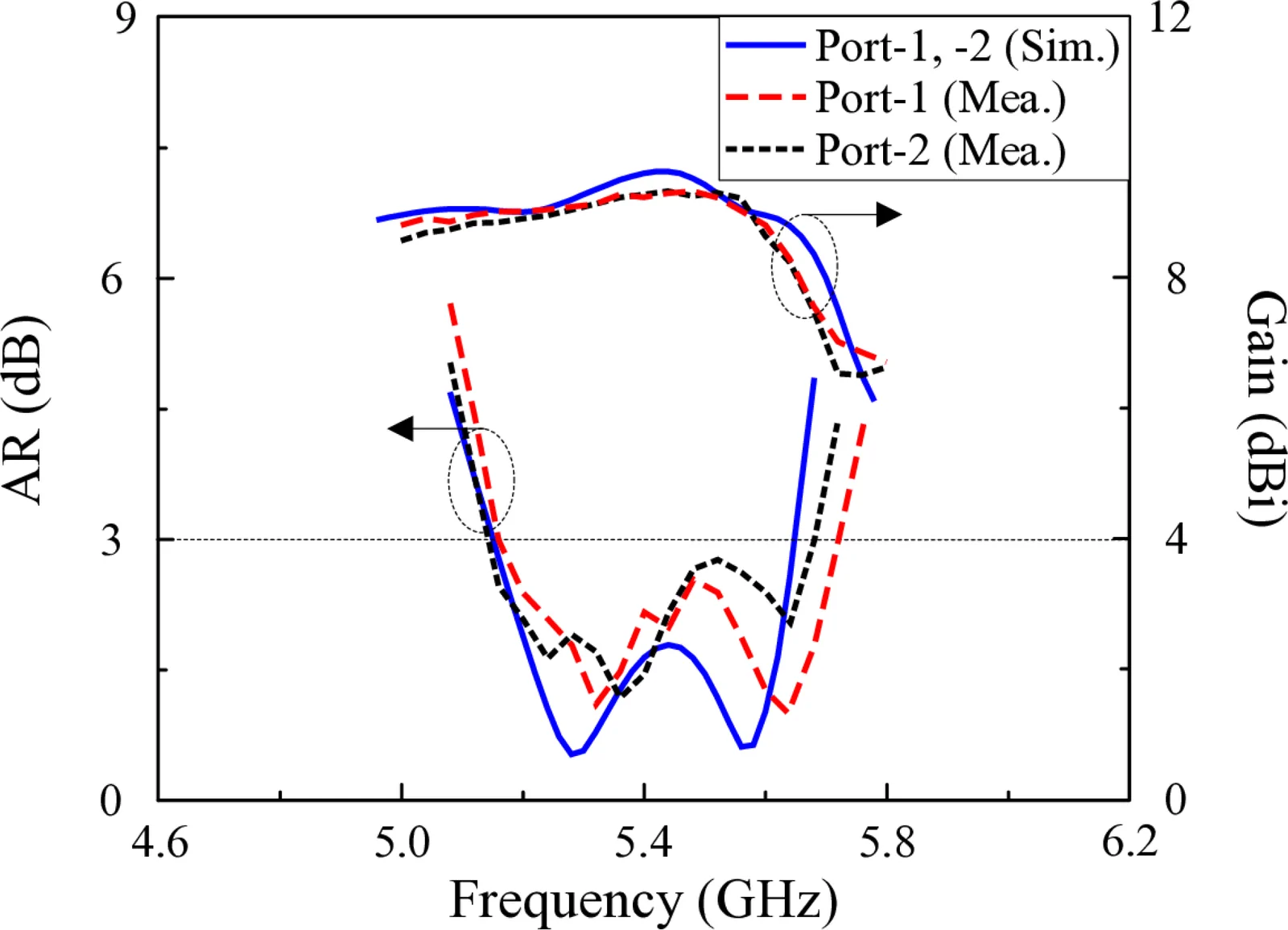
Simulated and measured AR and peak realized gain of the proposed antenna.

Fig 14 shows the simulated and measured radiation patterns of the proposed antenna at different frequencies in the operating BW. Both co- and cross-polarization radiation patterns are considered. The data indicates that the proposed antenna strongly radiates left-hand CP (LHCP). The antenna shows good directional radiation patterns at both frequencies. The back radiation is about 15 dB lower than the forward radiation, confirming a good front-to-back ratio.

**Fig 14:**
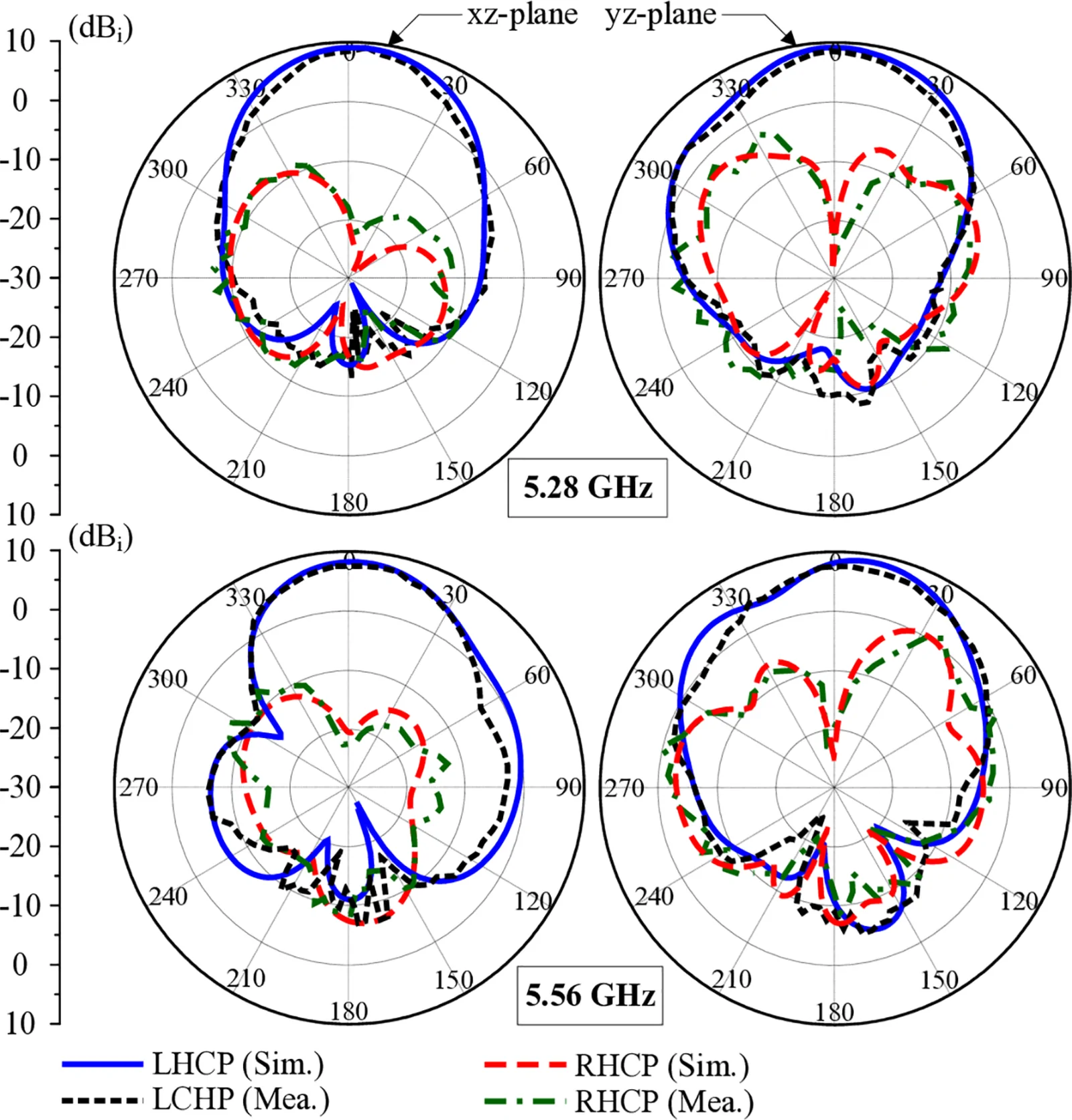
Simulated and measured radiation patterns of the proposed antenna.

Although experimental validation on an actual UAV platform has not yet been conducted, the proposed antenna exhibits several characteristics that make it well suited for UAV communication systems. First, its simple single-layer configuration facilitates low-profile integration onto UAV platforms. Second, the CP radiation helps mitigate polarization mismatch caused by changes in UAV orientation, such as pitching and rolling. Third, the measured peak gain of 9.4 dBi, together with an approximately 15-dB front-to-back ratio, concentrates the radiated energy toward the intended link direction, which is beneficial for improving link coverage and communication range. Finally, the MIMO configuration provides spatial diversity and supports enhanced link capacity in multipath propagation environments.

### MIMO diversity performance

In multipath-rich conditions and during dynamic flights of a UAV with constant pitching and rolling, the analysis of diversity characteristics of the suggested antenna is essential to achieve reliable data transfer with higher rates of data throughput. The spatial independence and isolation of the proposed system are evaluated according to three widely used indicators: Envelope Correlation Coefficient (ECC), Channel Capacity Loss (CCL), and Mean Effective Gain (MEG), which can be determined by the following equations.


$$\begin{array}{c} ECC=\frac{|S_{11}^{*}S_{12}+S_{21}^{*}S_{22}{|}^{2}}{\left(1-|S_{11}{|}^{2}-|S_{21}{|}^{2})(1-|S_{22}{|}^{2}-|S_{12}{|}^{2}\right)} \end{array}$$ (1)

$$\begin{array}{c} CCL=-\log_{2}\det(\Psi^{R}) \end{array}$$ (2)

where $\Psi^{R}$ is the correlation matrix formed by components $\rho_{ii}=1-(|S_{ii}{|}^{2}+|S_{ji}{|}^{2})$ and $\rho_{ij}=-(S_{ii}^{*}S_{ij}+S_{ji}^{*}S_{jj})$ for i,j∈{1,2}.


$$\begin{array}{c} MEG_{i}=0.5\left(1-\sum_{j=1}^{2}|S_{ij}{|}^{2}\right) \end{array}$$ (3)

where ECC represents the correlation value of radiation patterns of two ports; ECC should equal zero in an ideal situation, while satisfactory diversity of radiation pattern is characterized as ECC < 0.5. CCL represents the capacity to receive information without loss in data rate; the maximum level of CCL should be < 0.4 bits/s/Hz. Furthermore, MEG represents the reception gain that can be achieved by an antenna in fading conditions compared to an isotropic antenna. High performance antennas require the individual MEG value for each port between −3 dB and −6 dB, in which the ideal level should be as close to −3 dB as possible. The measured results for those metrics are displayed in Fig 15. Across the measured CP band of 5.16–5.68 GHz, the ECC remains close to zero, the CCL stays below the commonly adopted 0.4 bit/s/Hz criterion, and the MEG values of the two ports are comparable and close to the desirable −3 dB level. As these are widely used MIMO performance criteria, the results support the suitability of the proposed antenna for multipath UAV communication environments..

**Fig 15:**
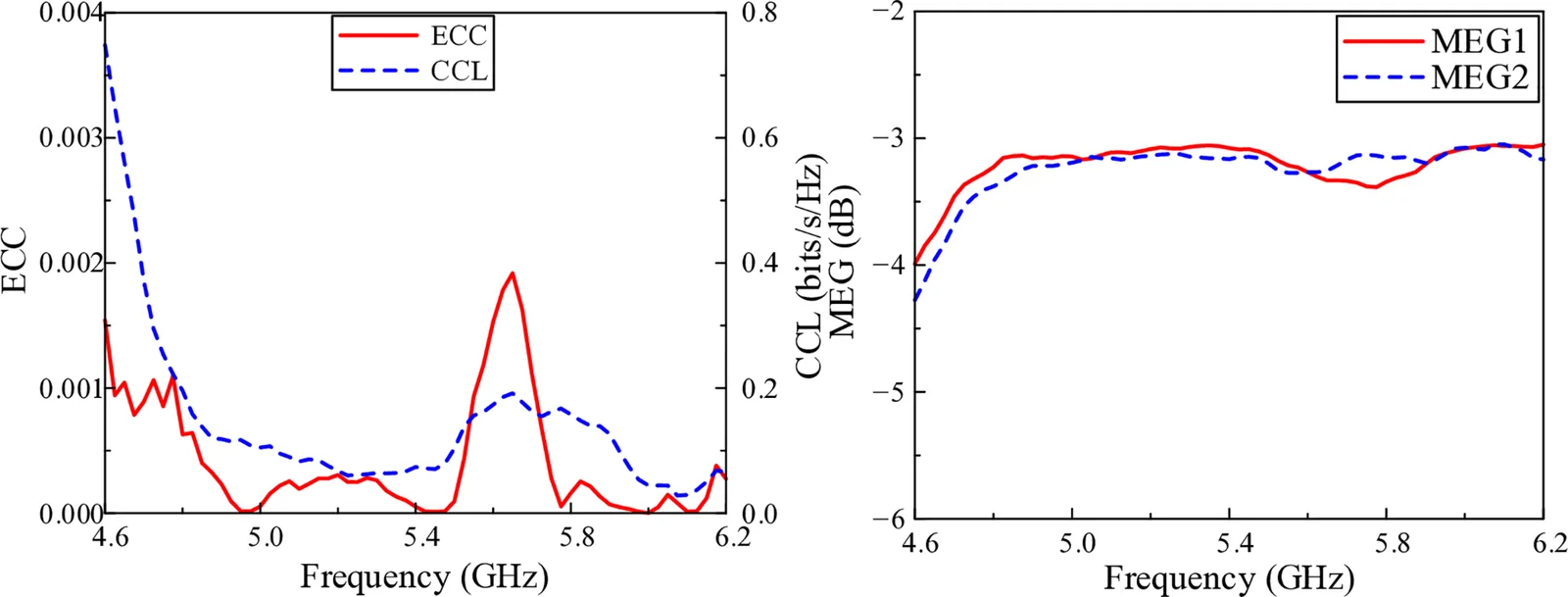
Measured diversity performance of the proposed antenna.

### Performance comparison

The performance comparison of two-port CP MIMO antennas is summarized in Table 1. It is evident that the proposed antenna achieves the lowest profile among the compared designs. In addition, its single-layer configuration, high gain, and high front-to-back ratio (FBR) further highlight the advantages of the proposed design. The antennas reported in [18] and [19] are structurally more complex due to their multilayer configurations and additional power dividers. The designs in [16] and [21] exhibit lower gain than the proposed antenna. Although the single-layer designs in [23–25] offer wider operating bandwidths, they have significantly higher profiles. Moreover, their use of slot-fed structures typically results in increased back radiation and, consequently, a lower FBR.

**Table 1:** Performance comparison among wideband 2-port CP MIMO antennas.

Ref.	Iso. (dB)	Gain (dBic)	BW (%)	FBR (dB)	Overall dimension (λ)	No. of layers	DGS	Power divider	Design complexity
[16]	30	8.5	11.1	13	1.36×0.94×0.05	2	Yes	No	High
[18]	20	8.0	13.7	10	2.14×1.07×0.42	4	No	Yes	Very High
[19]	24	9.9	47.9	10	1.31×1.12×0.02	2	No	Yes	Very High
[21]	26	6.0	14.5	15	0.92×0.73×0.05	2	No	No	Medium
[23]	30	11.0	16.8	25	1.70×0.85×0.05	1	No	No	Low
[24]	20	8.0	11.9	10	1.55×0.88×0.06	1	Yes	No	Medium
[25]	20	8.5	25.6	9	1.44×0.80×0.06	1	Yes	No	Medium
**Prop.**	**19**	**9.4**	**9.6**	**15**	1.80×0.95×0.02	**1**	**No**	**No**	**Low**

## Conclusion

This paper presents a wide band, high gain, CP MIMO antenna design for use in UAV applications. In order to cope with the problem of polarization mismatch and multipath fading effects that occur while flying, this design utilizes a highly stable CP radiation technique. In addition, the bulky design is addressed by employing a novel approach using only one layer planar structure. It consists of a 4×4 arrangement made up of 16 truncated corner patches fed centrally by a single rectangle patch. The measured results show that the design of a 2-port MIMO operates within 9.6% frequency range of 5.16 GHz to 5.68 GHz, with an isolation greater than 19 dB and a peak broadside gain of 9.4 dBi. This compact, efficient geometry provides a potential solution for stable, high-speed UAV wireless communications.
