# Supplementary material for: Single-layer low-profile high-gain wideband circularly polarized MIMO antenna for UAV applications
Source: PLoS One. 2026 Nov 13;21(11):e0362484. doi: 10.1371/journal.pone.0362484 (PMC13766285; doi:10.1371/journal.pone.0362484)
Supplement: Supplementary file 1 [file pone.0362484.s002.pdf]

Submission ID: PONE-D-26-34262

Original Article Title: "Single-Layer Low-Profile High-Gain Wideband Circularly Polarized MIMO Antenna for UAV Applications"

To: Reviewer

Re: Response to reviewer

Dear Reviewer,

We sincerely thank you for the time and effort devoted to reviewing our manuscript and for the constructive comments. The comments have helped us improve the technical clarity, presentation, and discussion of the proposed antenna. We have carefully considered each point and provide our point-by-point responses below.

The revised manuscript incorporates the corresponding clarifications where they are supported by the available results. Changes in the revised manuscript are highlighted in red for convenient identification.

Best regards,

<u>Reviewer 1:</u> *The manuscript is well-organized and maintains the reader's interest. However, several important concerns should be addressed before the paper can be considered.*

Concern #1: *The authors should more clearly articulate the motivation behind this work. Recently published studies on MIMO antennas have introduced novel structures, and the authors are encouraged to compare their works.*

Author response: The authors sincerely thank the Reviewer for this important comment. The motivation of this work is to simultaneously satisfy four requirements that are particularly relevant to long-range UAV links: wide circular-polarization (CP) bandwidth, high directional gain, low-profile single-layer implementation, and MIMO operation with adequate isolation.

Existing monopole/slot CP MIMO antennas are generally low gain and bidirectional, whereas conventional patch-based CP MIMO antennas usually provide narrow CP bandwidth. Wider-band and higher-gain solutions often rely on stacked layers, superstrates, power dividers, or DGSs, which increase the profile, structural complexity, or back radiation. The proposed design is therefore intended to provide a balanced solution using a single-layer capacitively coupled patch aperture. Noted that to the best of authors' knowledge, all relevant works have been cited in this paper and comparison Table 1 as well.

Author action: The motivation is clarified in Paragraph 1, Section "Wideband High-Gain CP Antenna" of the revised manuscript.

Concern #2: *It is unclear why this particular resonator structure was chosen. Considering that wideband antennas already exist covering the targeted frequency bands, please clarify the rationale for this design choice.*

Author response: The selected resonator arrangement is motivated by the need to enhance both CP bandwidth and gain without introducing additional layers or a corporate feeding network. A truncated-corner patch supports the two near-degenerate orthogonal modes required for CP radiation. By incorporating additional truncated-corner patches with slightly different resonant dimensions, a second CP resonance is introduced at a nearby higher frequency, thereby broadening the 3-dB axial-ratio bandwidth. Extending this concept to a 4×4 capacitively coupled aperture enlarges the effective radiating aperture and improves the broadside gain, while the central rectangular feed maintains a single-layer configuration.

Compared with the monopole/slot-based CP MIMO antennas in [6]–[10], which generally provide wide bandwidth but relatively low gain and bidirectional radiation, the proposed resonator offers higher directional gain. Conventional microstrip patch antennas [11]–[15] typically exhibit narrow CP bandwidths ($<3\%$) and moderate gain of about 7 dBi. Wider bandwidth and higher gain have been achieved using additional radiators, power dividers, frequency selective structures (FSSs), or defected ground structures (DGSs) [16]–[21], but often at the expense of increased profile, structural complexity, or back radiation. Single-layer wideband CP designs with bandwidths above 12% have also been reported [22]–[25]; however, their gain generally remains below 9 dBi, and some designs exhibit increased back radiation due to utilization of DGS.

Overall, the adopted resonator arrangement provides a favorable combination of wide CP bandwidth, high directional gain, low-profile single-layer implementation, and MIMO operation with adequate isolation.

Author action: The reason for choosing the resonator structure is clarified in Paragraph 2, Section "Wideband High-Gain CP Antenna" of the revised manuscript.

Concern #3: *An equivalent circuit model should be provided to elucidate the working principle of the proposed antenna.*

Author response: The authors would like to thank the Reviewer for this constructive comment. It should be noted that the truncated-corner patch, which excites two near-degenerate orthogonal modes for CP radiation, is a well-established CP realization technique. In addition, the CP generation mechanism of the adopted aperture has been thoroughly investigated in [26].

The authors agree that an equivalent-circuit model could provide further insight into the operating characteristics of the proposed design. However, developing an accurate circuit model for the strongly coupled multi-resonator aperture would require considerable additional complexity. Therefore, the authors believe that full-wave electromagnetic simulation provides a more appropriate and reliable approach for analyzing the operating mechanism of the proposed antenna.

Concern #4: *The MIMO performance merits further exploration, including an assessment of communication capabilities, as demonstrated in studie.*

Author response: The MIMO communication capability is evaluated using both inter-port isolation and standard diversity metrics. Across the measured CP band of 5.16–5.68 GHz, the isolation remains better than 19 dB. In addition, ECC, CCL, and MEG are examined to assess port correlation, channel-capacity degradation, and diversity performance. The ECC remains close to zero, the CCL stays below the commonly adopted 0.4 bit/s/Hz criterion, and the MEG values of the two ports are comparable and close to the desirable −3 dB level. As these are widely used MIMO performance criteria, the results support the suitability of the proposed antenna for multipath UAV communication environments.

Author action: Further discussion about the MIMO performance is added to Paragraph 2, Subsection "MIMO diversity performance", Section "Measurement results" of the revised manuscript.

Concern #5: *Inclusion of a comparison table contrasting this work with existing designs is necessary to highlight the improvements and distinctions.*

Author response: The authors agree. Overall, the pros and cons of the proposed work in comparison with the related works can be summarized as follows:

Advantages:

- Single-layer and very low-profile structure.
- High gain with good directional radiation/FBR.
- No DGS, FSS or power divider, reducing complexity.

Disadvantages:

- Limited CP bandwidth and isolation.
- Relatively large planar footprint.

Author action: The pros and cons of the proposed design are summarized in Paragraph 2, Subsection "Performance comparison", Section "Measurement results" of the revised manuscript.

Concern #6: *The authors must explicitly emphasize the novelty and key contributions of their work.*

Author response: The novelty of the work lies in the combination of the following features within one planar layer: (i) a centrally excited 4 × 4 truncated-corner patch aperture for high gain; (ii) multiple slightly different CP resonators that create adjacent AR resonances and broaden the CP operating band; (iii) capacitive excitation that avoids a multilayer corporate feed; and (iv) a two-port MIMO configuration achieving measured 5.16–5.68 GHz CP operation, isolation better than 19 dB, and peak gain of 9.4 dBi.

The contribution is therefore the simultaneous realization of wideband CP, high gain, low profile, and simple MIMO integration rather than any single metric alone.

Author action: The contribution of the proposed design is emphasized in Paragraph 6, Section "Introduction" of the revised manuscript.

Concern #7: *The manuscript's language requires polishing to enhance clarity and readability.*

Author response: Agree.

Author action: The manuscript is thoroughly revised.

Concern #8: *Figures should be improved for better quality and clarity.*

Author response: Agree.

Author action: All figures quality is improved in the revised manuscript.

Reviewer 2: *This article is not showing any novelty with respect to the design, modeling and analysis.*

Concern #1: *Since the antenna is claimed to be single-layer, the authors should provide a detailed explanation of how high gain, wide impedance bandwidth, circular polarization, and adequate MIMO isolation are simultaneously achieved without multilayer structures, additional superstrates, or complex feeding networks.*

Author response: The authors would like to thank the Reviewer for this constructive comment. Further explanation of how wideband operation, high gain, and adequate MIMO isolation are simultaneously achieved is provided below.

First, the wide CP bandwidth is realized by combining two nearby CP resonances. The four central truncated-corner patches mainly generate the lower CP resonance, whereas the surrounding patches, with slightly different dimensions, introduce an additional CP resonance at a higher frequency. The overlap of these resonances broadens the 3-dB AR bandwidth. Further discussion is provided in the response to Concern #2.

Second, the gain enhancement is achieved by enlarging the active radiating aperture. The antenna gain is related to the effective radiating aperture according to $G = 4\pi A_e/\lambda^2$. Although Designs 1–3 have similar overall dimensions, Design-1 contains only the four central radiating patches, whereas Design-3 employs additional surrounding patches. The current distributions in Fig. 1R show that, at both CP resonances, significant currents are distributed over a larger portion of the aperture. Consequently, Design-3 provides a larger effective radiating region and achieves higher gain. This is also confirmed by the simulated results, where the gain increases from approximately 8.0 dBi for Design-1 to 9.8 dBi for Design-3. Further extension to a 5 × 5 arrangement provides only limited gain improvement because the outermost patches are weakly excited.

Finally, the MIMO isolation is achieved primarily through spatial separation between the two radiating apertures. The optimized center-to-center spacing of 50 mm provides measured isolation better than 19 dB across the CP operating band without requiring a DGS or additional decoupling network. Therefore, the proposed configuration simultaneously achieves wideband CP operation, high gain, and adequate MIMO isolation while preserving a single-layer structure without superstrates or complex feeding networks.

Author action: Further discussion about the antenna operation characteristic is added to Paragraphs 3 and 4, Subsection "Design-3 with enhanced AR BW and gain radiation", Section "Wideband High-Gain CP Antenna" of the revised manuscript.

Concern #2: *The physical mechanism responsible for the wideband circular polarization should be explained rigorously. Current-mode/electric-field/current-distribution analysis at the relevant resonant frequencies should be provided to demonstrate how the orthogonal modes are generated and how their phase difference approaches 90°.*

Author response: The authors would like to thank the Reviewer for this constructive comment. Fig. 1R presents the current distributions of Design-3 at the two CP resonances, 5.3 and 5.5 GHz. At both frequencies, two orthogonal field components are observed, and the current rotates clockwise as the phase changes from 0°to 90°, confirming LHCP radiation.

In addition, the 5.3-GHz resonance is mainly associated with strong currents on the central patches, whereas the 5.5-GHz resonance shows stronger current distribution on the outer patches. This clearly demonstrates the role of the outer patches in generating additional AR resonance.

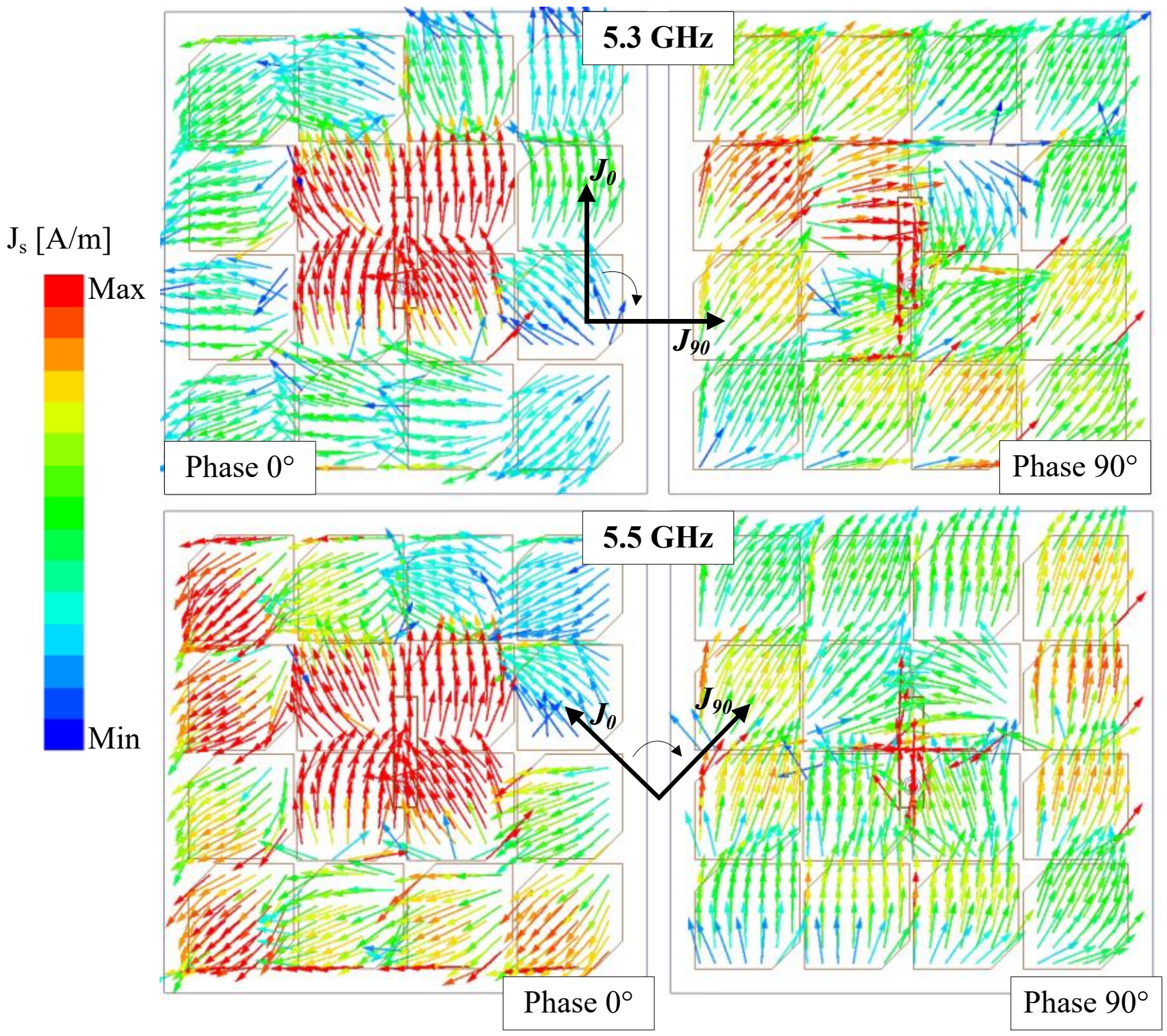

Fig. 1R. Simulated current distributions of Design-3 at different frequencies.

Author action: A discussion about the current distribution is added to Paragraph 3, Subsection "Design-3 with enhanced AR BW and gain radiation", Section "Wideband High-Gain CP Antenna" of the revised manuscript.

Concern #3: *The authors should clarify whether the enhancement originates from parasitic elements, slots, a modified ground plane, array effects, or another mechanism.*

Author response: The performance enhancement originates mainly from the capacitively coupled parasitic patch aperture, rather than from slots or a modified ground plane. The truncated corners are local perturbations used to generate the two orthogonal CP modes. Patches of different dimensions provide adjacent CP resonances for AR-bandwidth enhancement, while increasing the number of radiating patches enlarges the effective aperture and improves the broadside gain. The ground plane remains continuous, and no DGS are employed. Likewise, no multilayer superstrate or external power divider is required.

Author action: The original enhancement is discussed in Paragraph 2, Subsection "Design-3 with enhanced AR BW and gain radiation", Section "Wideband High-Gain CP Antenna" of the revised manuscript.

Concern #4: *The motivation for using this antenna specifically for UAV applications should be strengthened. The authors should discuss UAV-specific requirements such as low profile, low weight, aerodynamic integration, polarization stability, platform compatibility, communication range, and resistance to installation-induced detuning.*

Author response: The UAV motivation is based on several system-level requirements as follows:

- The single-layer and simple structure is favorable for integration on a UAV surface.

- CP radiation mitigates polarization mismatch caused by UAV pitching and rolling.
- The measured peak gain of 9.4 dBi and approximately 15-dB front-to-back ratio concentrate energy toward the intended link direction and are beneficial for extending communication range.
- MIMO operation supports higher link capacity in multipath environments.

However, experimental validation on a UAV platform has not yet been conducted. In response to the Reviewer's valuable suggestion, the authors have identified platform-mounted validation as an important direction for future work.

Author action: The suitable of the proposed design for UAV application is discussed in Paragraph 3, Subsection "Antenna performance", Section "Measurement results" of the revised manuscript.

Concern #5: *The comparison table should be substantially improved. It should include recent literature and compare frequency range, fractional bandwidth, 3-dB AR bandwidth, peak gain, efficiency, isolation, ECC, number of ports, dimensions, normalized electrical size, profile, and fabrication complexity. A simple comparison of gain and bandwidth is not sufficient.*

Author response: The authors agree that a more comprehensive comparison is necessary. In response to the Reviewer's comment, the following parameters have been considered:

- **Operating bandwidth:** For CP antennas, the operating bandwidth is defined as the frequency range where $|S_{11}| < -10$dB and AR < 3 dB. Since the operating frequency can be shifted by scaling the antenna dimensions, the authors believe that reporting the operating bandwidth is sufficient, particularly given the limited table space and the need to include other important parameters.
- **Gain and isolation:** The peak gain and minimum isolation have already been included in the comparison table.
- **Efficiency:** Radiation efficiency is not reported in many of the referenced works; therefore, a consistent comparison cannot be made.
- **ECC:** The ECC values of the compared designs are generally well below the commonly accepted criterion. Therefore, this parameter provides limited additional differentiation and has not been included.
- **Number of ports:** To ensure a fair comparison, Table 1 is limited to two-port MIMO antennas.
- **Antenna size:** The antenna size is already included in the "Overall Dimension" column and is expressed in terms of normalized electrical dimensions rather than physical dimensions.
- **Structural complexity:** The authors agree that complexity is an important comparison factor. Relevant indicators, including the number of layers, use of DGS, and requirement for a power divider, have therefore been included in the revised comparison table.

Author action: The comparison Table 1 is updated with new metric.

Concern #6: *The complete antenna geometry, dimensions, coordinate definitions, substrate information, and feeding configuration should be provided. The present design should be sufficiently detailed so that another researcher can reproduce the antenna without ambiguity.*

Author response: Agree.

Author action: More detailed information is added to Paragraph 1, Subsection "Antenna geometry", Section "2-Port Wideband High-Gain CP Antenna" of the revised manuscript.

Concern #7: *Major Revision is recommended. The concept appears potentially useful for UAV communication; however, the manuscript requires stronger evidence of novelty, circular-polarization mechanism, MIMO decoupling, wideband operation, gain enhancement, UAV-platform suitability, and experimental validation before the technical contribution can be considered fully established.*

Author response: The authors sincerely thank the Reviewer for the detailed and constructive assessment. The manuscript has been carefully revised in accordance with the Reviewer's comments. We hope that the revisions have satisfactorily addressed the concerns and that the manuscript is now suitable for publication.
