# Supplementary material for: Single-layer low-profile high-gain wideband circularly polarized MIMO antenna for UAV applications
Source: PLoS One. 2026 Nov 13;21(11):e0362484. doi: 10.1371/journal.pone.0362484 (PMC13766285; doi:10.1371/journal.pone.0362484)
Supplement: Supplementary file 2 [file pone.0362484.s003.docx]

**Submission ID:** PONE-D-26-34262

**Original Article Title:** “Single-Layer Low-Profile High-Gain Wideband Circularly Polarized MIMO Antenna for UAV Applications”

**To:** Reviewer

**Re:** Response to reviewer

Dear Reviewer,

We sincerely thank you for the time and effort devoted to reviewing our manuscript and for the constructive comments. The comments have helped us improve the technical clarity, presentation, and discussion of the proposed antenna. We have carefully considered each point and provide our point-by-point responses below.

The revised manuscript incorporates the corresponding clarifications where they are supported by the available results. Changes in the revised manuscript are highlighted in red for convenient identification.

Best regards,

**Reviewer 1:** *This paper is well organized, and can be accepted. One minor can be considerated.
The motivation can be clearly articulated.*

**Author response:** The authors would like to thank the Reviewer for the constructive comment.

**Author action:** A motivation is strengthen in Paragraph 5, Section “Introduction” of the revised manuscript.

**Reviewer 2:** *Satisfied with the responses and revision. Authors presented the technical content in the satisfactory mode.*

**Author response:** The authors would like to thank the Reviewer for your valuable time spent on reviewing our submission.
